# Measurement Properties of the Modified Incremental Step Test for Assessing Exercise Capacity in Individuals With Type 2 Diabetes

**DOI:** 10.1002/pri.70224

**Published:** 2026-04-23

**Authors:** Larissa Barbosa de Carvalho, Renata Cruzeiro Ribas, Maria Clara Noman de Alencar, Danielle Aparecida Gomes Pereira, Patrícia Fernandes Trevizan, Lilian Pinto da Silva

**Affiliations:** ^1^ Graduate Program in Rehabilitation Sciences and Physical‐Functional Performance Federal University of Juiz de Fora Juiz de Fora Minas Gerais Brazil; ^2^ Graduate Program in Rehabilitation Sciences Department of Physical Therapy Federal University of Minas Gerais Belo Horizonte Minas Gerais Brazil; ^3^ OnzeMets Reabilitação Prevenção Cardiovascular e Ensino Belo Horizonte Minas Gerais Brazil

**Keywords:** cardiorespiratory fitness, diabetes mellitus, exercise test, validation study

## Abstract

**Background and Purpose:**

A reduction in exercise capacity (EC) can be observed in individuals with type 2 diabetes (T2D) on cardiopulmonary exercise test (CPET). However, CPET is a costly maximal exercise test, limiting its routine use. This study aimed to analyze the criterion validity, convergent validity, and test‐retest reliability of the Modified Incremental Step Test (MIST), a simple, inexpensive, and portable submaximal exercise test, for assessing EC in individuals with T2D.

**Methods:**

Fifty individuals with T2D (56.9 ± 11.4 years old, 50% male) attended two visits within a 2‐ to 10‐day interval. On the first visit, participants underwent a CPET. On the second visit, two repetitions of the submaximal exercise tests, the MIST and the 6‐minute walk test (6MWT), were conducted in a random order. Criterion validity was assessed using the Pearson correlation coefficient between the number of steps climbed in the best‐performed MIST and peak oxygen consumption (peak VO_2_) from CPET. Convergent validity was evaluated by the Pearson correlation coefficient between the best MIST performance and the distance covered in the best 6MWT. Test–retest reliability was determined using the intraclass correlation coefficient (ICC) between two MIST repetitions.

**Results:**

Convergent validity was supported by a moderate correlation between the number of steps climbed in the best‐performed MIST and the distance covered in the best 6MWT (*r* = 0.65; *p* < 0.001; *n* = 50). In contrast, criterion validity (*r* = 0.53; *p* = 0.023; *n* = 18) and test‐retest reliability (ICC [95% CI] = 0.64 [0.44–0.78]) did not meet the criteria for adequate measurement properties.

**Discussion:**

This multicenter methodological study, conducted in accordance with COSMIN recommendations, contributes to the literature by evaluating the psychometric properties of the MIST for assessing EC in individuals with T2D. Although the findings do not support the MIST as a substitute for CPET, its convergent validity may support its use when 6MWT execution is not possible.

## 1 Introduction

Individuals with type 2 diabetes (T2D) show reduced exercise capacity (EC) (Macedo et al. 2023), which increases the risk of mortality (Wei et al. 2000) and is associated with a higher number of cardiovascular events in this population (Seyoum et al. 2006; Zafrir et al. 2015). In individuals with T2D, impairments in cardiac function, peripheral vascular resistance, autonomic regulation, muscle fiber composition, chronic inflammation, and insulin resistance may lead to elevated blood pressure responses, reduced perfusion and oxygen diffusion, metabolic imbalance, and decreased muscle strength, ultimately contributing to tissue hypoxia, early fatigue, and reduced EC (Bilak et al. 2021; Espino‐Gonzalez et al. 2024; Nesti et al. 2020). Thus, assessing EC becomes relevant as part of the clinical follow‐up of individuals with T2D.

The cardiopulmonary exercise test (CPET) is the gold standard for assessing EC through the direct measurement of peak oxygen uptake (peak VO_2_), which allows investigation of cardiopulmonary and metabolic responses during exercise (ACSM 2021). However, CPET is costly and requires qualified professionals and specific equipment, limiting its availability (Neder et al. 2021).

Considering this, simple and inexpensive submaximal exercise tests aim to provide reliable and reproducible alternatives to CPET for assessing EC in different populations. Although they are less accurate than maximal exercise tests, parameters from submaximal exercise tests (e.g., end‐test HR or average walking speed), combined with Rating of Perceived Exertion (RPE), can guide moderate aerobic training prescription (Hansen et al. 2025). Additionally, since submaximal exercise tests offer a lower risk of adverse cardiovascular events, depending on the cardiovascular risk of the target population, their execution does not require physician supervision and consequently brings high applicability to physical therapists in clinical settings (Sartor et al. 2013). To date, the only submaximal exercise test with adequate measurement properties to assess EC in individuals with T2D is the six‐minute walk test (6MWT). The distance walked correlates significantly with peak VO_2_ from CPET (Nolen‐Doerr et al. 2018) and with metabolic equivalents (METs) from the maximal treadmill test (Ramírez Meléndez et al. 2019). However, the 6MWT has limitations: (1) it requires a large, unobstructed space; (2) it depends on favorable weather when done outdoors; and (3) its self‐paced nature may allow motivation to influence results (Halliday et al. 2020).

Submaximal exercise tests using a step ergometer have been increasingly used due to their minimal space requirements, allowing EC assessment in hospital rooms and home settings (José et al. 2021). The modified incremental step test (MIST) is an externally paced incremental step test that can elicit maximal physiological responses in adults with chronic obstructive pulmonary disease (COPD) (Dal Corso et al. 2013; De Andrade et al. 2012). Some studies have evaluated the psychometric properties of the MIST for EC assessment in individuals with pulmonary diseases, such as pulmonary hypertension (Vieira et al. 2020), COPD (King et al. 2023), and asthma (Barbosa et al. 2022). However, only the study by Barbosa et al. (2022) had an adequate sample size according to the COSMIN recommendations (Mokkink et al. 2012). Additionally, to date, no studies have used MIST to assess EC in individuals with cardiometabolic diseases such as T2D.

This study aimed to analyze the measurement properties of the MIST—criterion validity, convergent validity, and test–retest reliability—to determine whether it could serve as an inexpensive and portable alternative for assessing EC in individuals with T2D. Additionally, floor and ceiling effects and the learning effect of the MIST were analyzed, as well as cardiovascular responses and RPE were compared between MIST, 6MWT, and CPET.

## 2 Methods

### 2.1 Study Design and Ethical Considerations

This is a methodological study conducted across two research centers, Federal University of Minas Gerais (UFMG) and Federal University of Juiz de Fora (UFJF), and developed following the COSMIN recommendations (Mokkink et al. 2012). The study protocol was approved by the ethics committees of both centers, and all participants signed informed consent before inclusion.

### 2.2 Participants

Individuals with self‐reported T2D confirmed using oral antidiabetic agents and/or insulin, aged ≥ 18 years, both sexes, were recruited by convenience from an interested participant list in an ongoing multicenter study (Silva et al. 2024), and via dissemination in public/private healthcare, social media, and university staff at the two research centers. Eligibility criteria included: (1) no mobility, cognitive, hearing, and/or visual impairments that would prevent performance of the submaximal exercise tests; (2) absence of intermittent claudication; (3) no unstable coronary artery disease and/or heart failure; and (4) no complex cardiac arrhythmias. Participants were excluded if they failed to attend the second visit; were unable to complete the submaximal exercise tests and/or the CPET due to persistent hypo‐ or hyperglycemia (capillary blood glucose < 90 mg/dL or > 250 mg/dL (SBD 2020)); did not meet the criteria for maximal CPET (Carvalho et al. 2024); presented clinical or electrocardiographic changes requiring test interruption; or if CPET could not be completed due to technical issues.

### 2.3 Visits

Participants were required to attend two visits within a 2‐ to 10‐day interval. At the first visit, eligible individuals were informed about all research procedures, signed the informed consent form, and underwent an initial assessment to collect demographic, anthropometric, and clinical data. Additionally, participants from the UFMG center were submitted to the CPET during this visit. At the second visit, the submaximal exercise tests (6MWT and MIST) were conducted in a randomized order. An assistant investigator generated a random test sequence using an online tool, and the principal investigator (PI) stored it in a password‐protected file. The randomization information was provided by the PI to the assessor only when the assessments were about to start to ensure sequence concealment. The capillary blood glucose was measured at the beginning and end of the visit.

### 2.4 Cardiopulmonary Exercise Test (CPET)

The CPET was conducted by an experienced cardiologist and a trained researcher using a gas analyzer (Medical Graphics CPX Ultima, Miami, FL, USA) and a treadmill ergometer (Millenium Classic CI, Inbramed/Inbrasport, Brazil), with a ramp incremental protocol (Carvalho et al. 2024) and simultaneous 12‐lead electrocardiogram recording. Blood pressure (BP), heart rate (HR), and RPE, measured by the modified Borg scale (Borg 1982), were monitored before, during, and after the CPET. The capillary blood glucose was measured before and after CPET. The ramp protocol was fully individualized in terms of speed and grade (initial and final) based on the upper limit of physical exertion defined as maximal for each participant, and the target workload was set to achieve a test duration from 8 to 12 min. This upper limit was established based on participants' clinical and physical characteristics and self‐reported level of physical activity.

The following sequence of procedures was adopted in all CPETs: (1) Three‐minutes resting standing on the treadmill with electrocardiogram and gas exchange monitoring; (2) two‐minutes warm‐up walking at comfortable speed without grade; (3) ramp incremental protocol fully variable with increments distribution over 12‐min; (4) one‐minute active recovery walking at speed of 2.5 km/h without grade; and (5) five‐minutes passive recovery seated with continuous electrocardiogram monitoring. The passive recovery could be extended if necessary (symptoms, inadequate decrease of BP, persistent tachycardia).

The procedures to calibrate flow/volume and gas analyzers, as well as the criteria to determine a maximal test, and to conclude or interrupt the test, followed the Brazilian Guidelines on Exercise Testing in the Adult Population (Carvalho et al. 2024). The EC was determined by peak VO_2_ (L/min) measured at maximal effort intensity (Skinner and Mclellan 1980).

### 2.5 Submaximal Exercise Tests

#### 2.5.1 Modified Incremental Step Test (MIST)

The MIST consists of stepping up and down a single 20‐centimeter step at a progressive pace, following an auditory signal, starting at 10 steps per minute and increasing by one step every 30 s (Dal Corso et al. 2013; de Andrade et al. 2012). The MIST was conducted by trained researchers, and prior to its execution, participants underwent a one‐minute familiarization to ensure proper interpretation of the auditory cues. HR, BP, and RPE were measured immediately before and after the test while standing. The MIST was terminated when participants were unable to keep pace with the auditory signal within 15 s or was interrupted if (1) participants presented any symptom of exercise intolerance (severe dyspnea and/or lower limb fatigue) (De Andrade et al. 2012); or (2) if participants reached 85% of the age‐predicted maximum HR (Jones 1975) as a safety criterion, given that individuals with cardiometabolic conditions such as T2D have a higher risk of cardiovascular disease (Sattar et al. 2023) and cardiac arrhythmias (Rawshani et al. 2023). The MIST was performed twice consecutively for test‐retest reliability analysis, with the interval between attempts lasting the time necessary for HR, BP, and RPE levels (Borg 1982) to return to baseline values, which was ≤ 30 min.

If the use of upper limb support was necessary to maintain participants' balance during the first test execution, the second execution was performed in the same manner (Dal Corso et al. 2013). The best‐performed MIST, defined as the highest number of steps completed, was considered for assessing EC, measured both by the absolute number of steps climbed and by the percentage of the predicted step count for each participant (Amaral et al. 2024).

#### 2.5.2 Six‐Minute Walk Test (6MWT)

The 6MWT was performed according to the American Thoracic Society/European Respiratory Society guidelines (American Thoracic Society 2002) and conducted by trained researchers. HR, BP, and RPE were measured immediately before and after the test in the standing position. The 6MWT was interrupted if participants showed any symptoms of exercise intolerance (severe dyspnea and/or lower limb fatigue) or if they reached 85% of the age‐predicted maximum HR (Jones 1975). The 6MWT was performed twice consecutively, with the interval between attempts lasting the time necessary for HR, BP, and RPE to return to baseline values, which was ≤ 30 min. The best‐performed 6MWT, defined as the greatest distance covered, was considered for assessing EC, measured both by distance in meters and by the percentage of the predicted distance for each participant (Britto et al. 2013).

### 2.6 Statistical Analysis

The sample size for evaluating criterion validity was estimated at 9 individuals, based on the correlation coefficient (*r* = 0.84) obtained in a previous study (Vieira et al. 2020), which was calculated using GPower 3.1 software with the following parameters: two‐tailed test, alpha level of 5%, statistical power of 95%, and an attrition rate of 15%. The sample size for evaluating convergent validity was set at 50 individuals following COSMIN recommendations (Mokkink et al. 2012).

Distribution of continuous data was assessed using the Shapiro–Wilk test. Data with a normal distribution were expressed as mean and standard deviation, while those without normal distribution were presented as median and interquartile range (25th–75th percentile). Categorical data were expressed as absolute and relative frequencies. A significant level of 5% was considered for all statistical tests. Statistical analyses were performed using SPSS v.20.0 (IBM Inc).

Criterion validity (Mokkink et al. 2012; Portney 2020) of the MIST was analyzed from the Pearson correlation coefficient obtained between the EC assessed by MIST and peak VO_2_ (L/min) assessed by CPET, while the convergent validity was analyzed from the Pearson correlation coefficient obtained between the EC assessed by MIST and by 6MWT. Considering that both MIST and 6MWT are submaximal exercise tests, the study hypothesis was that the correlation coefficient obtained between the EC assessed by MIST and by 6MWT would be moderately positive. Test–retest reliability of the MIST was analyzed using the intraclass correlation coefficient (ICC). Both correlation coefficients for criterion validity and ICC were considered adequate if ≥ 0.70 (Prinsen et al. 2016). The correlation coefficient for convergent validity was classified as: 0.00–0.25 (little or none), 0.25–0.50 (small), 0.50–0.75 (moderate), and > 0.75 (high to excellent) (Portney 2020).

The presence of floor and ceiling effects was examined by calculating the proportion of participants whose number of steps achieved in the MIST fell below the 10th percentile and above the 90th percentile, respectively (Bennett et al. 2002). Proportions below 15% were considered acceptable (Portney 2020). The number of steps climbed in the first and second MIST executions was compared using the paired *t*‐test, and the learning effect was analyzed by the mean difference between the test and retest. To compare HR and BP responses to the MIST with those elicited by the 6MWT and CPET, differences between values measured at the beginning and the end of the tests (deltas = *Δ*) were calculated. These, along with the final RPE and maximum HR achieved, were compared using the paired *t*‐test or Wilcoxon test (MIST vs. 6MWT, total sample) and one‐way ANOVA (MIST vs. 6MWT vs. CPET, subsample).

## 3 Results

**FIGURE 1:**
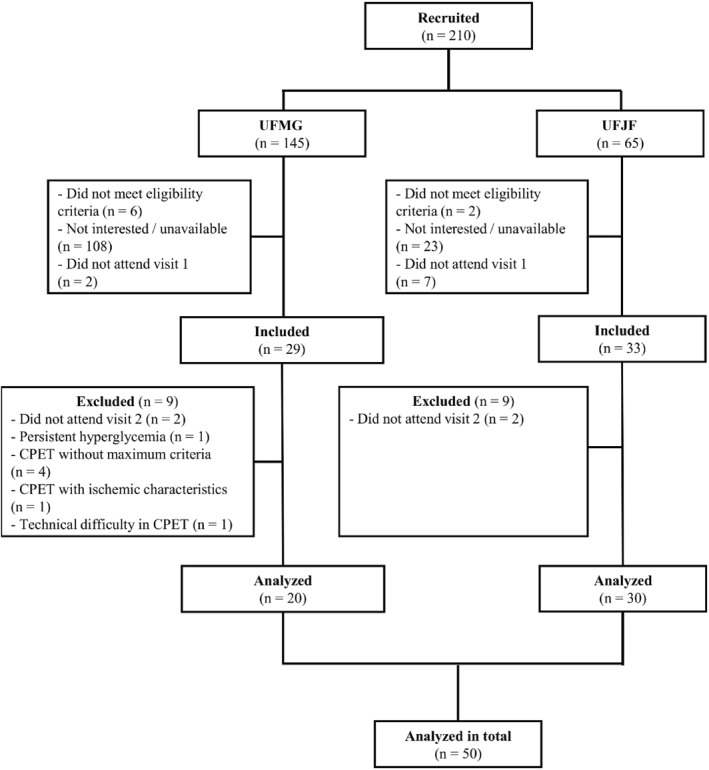
Flowchart of study participants.

Data were collected between October 2023 and March 2024. Figure 1 shows the study participant flowchart.

The total sample consisted of 50 participants who executed the submaximal exercise tests, and a subsample of 18 individuals who additionally were submitted to the CPET. Demographic, anthropometric, and clinical characteristics of the total sample and subsample, as well as information related to physical exercise and EC, are described in Table 1.

**TABLE 1:** Demographic, anthropometric, and clinical characteristics, physical exercise participation, and exercise capacity of the total sample submitted to submaximal exercise tests and the subsample submitted to the CPET.

Variables	Submaximal exercise tests (*n* = 50)	CPET (*n* = 18)
Demographic and anthropometric characteristics		
Age, years	56.9 ± 11.4	51.7 ± 9.9
Female—*n* (%)	25 (50)	10 (56)
Body mass index (kg/m^2^)	30.3 ± 5.4	29.5 ± 4.3
Clinical characteristics		
Time since diagnosis, years	10.2 ± 7.6	8.7 ± 4.6
Fasting glucose (mg/dL)	133.5 [110.8–172.8]^a^	133.5 [120.5–168.8]^b^
HbA1c (%)	6.9 [6.1–9.1]^a^	7.8 ± 1.4^b^
Oral antidiabetic—*n* (%)	47 (94)	18 (100)
Insulin therapy—*n* (%)	13 (26)	3 (17)
Diabetes complications—*n* (%)	10 (20)	3 (17)
Smoking—*n* (%)	1 (2)	0 (0)
Systemic arterial hypertension—*n* (%)	28 (56)	10 (56)
Dyslipidemia—*n* (%)	22 (44)	13 (72)
Physical exercise		
Exercise regularly—*n* (%)	29 (58)	9 (50)
Achieves exercise recommendations according to diabetes guidelines—*n* (%)	25 (50)	8 (44)
Exercise capacity		
VO_2_ Peak, L/min (% predicted)	—	1.9 ± 0.6 (88.0 ± 20.2)
Number of steps climbed on MIST (% predicted)	143.9 ± 68.2 (69.4 ± 24.6)	—
Distance covered on 6MWT, m (% predicted)	560.1 ± 83.5 (104.2 ± 13.2)	—

*Note:* a: *n* = 26; b: *n* = 8 (only results from blood tests dated until 90 days before the participant's enrollment date in the study were considered).

Abbreviations: 6MWT, 6‐min walk test; CPET, cardiopulmonary exercise testing; HbA1c, glycated hemoglobin; L/min, liters per minute; m, meters; MIST, modified incremental step test; VO_2_, oxygen consumption.

**FIGURE 2:**
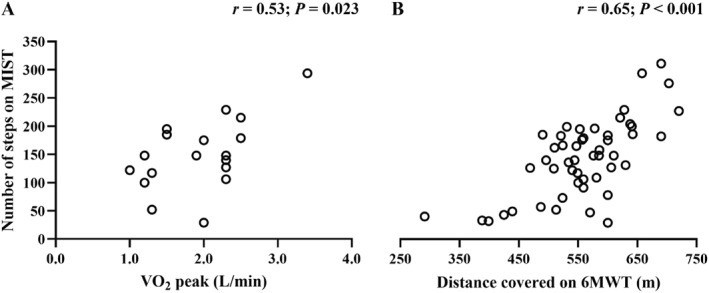
Scatter plots between (A) the number of steps climbed on MIST and peak VO_2_ (L/min) measured in the CPET (*n* = 18) and (B) the number of steps climbed on MIST and the distance covered on 6MWT (*n* = 50).

The number of steps climbed in the MIST showed a significant correlation with the VO_2_ (L/min) obtained in the CPET, with a correlation coefficient below the value required to confirm criterion validity, as illustrated in Figure 2 (panel A). Regarding convergent validity, the correlation between the number of steps climbed in the MIST and the distance covered in the 6MWT (meters) was significant and positive, with a moderate correlation coefficient, as illustrated in Figure 2 (panel B). The ICC (95% CI) was 0.64 (0.44–0.78), below the value required to confirm test‐retest reliability.

Five participants achieved a number of steps in the MIST below the 10th percentile (43.4 steps) or above the 90th percentile (225.8 steps), corresponding to a proportion of 10%, indicating the absence of floor or ceiling effects. No significant difference was observed between the number of steps climbed in the first and second MIST executions (121.3 ± 71.4 vs. 129.2 ± 60.7; *p* = 0.333). No learning effect was identified, with a mean difference between test and retest of −7.8 steps (95% CI: −23.8 to 8.2; *p* = 0.333). Cardiovascular responses and RPE to the submaximal exercise tests and CPET are presented in Table 2.

**TABLE 2:** Comparison of cardiovascular and rate of perceived exertion responses between submaximal exercise tests (total sample) and between the cardiopulmonary exercise test and submaximal exercise tests (subsample).

	Total sample (*n* = 50)			Subsample (*n* = 18)			
Variables	MIST	6MWT	*p*	CPET	MIST	6MWT	*p*
Δ HR. bpm	41.6 ± 16.0	33.2 ± 12.6	< 0.001	70.5 ± 22.5^a^	40.7 ± 15.6	34.4 ± 11.6	< 0.001
% Predicted maximum HR achieved	73.2 ± 9.9	68.4 ± 9.9	< 0.001	91.7 ± 10.8^a^	73.9 ± 9.2	71.7 ± 8.0	< 0.001
Δ SBP. mmHg	25.0 [12.0–40.0]	21.0 [10.8–30.5]	0.481	57.0 ± 22.6^a^	29.3 ± 19.4	25.1 ± 17.8	< 0.001
Δ DBP. mmHg	2.0 [−2.5–8.0]	0.0 [−2.0–2.5]	0.684	11.1 ± 10.5^a^	−1.2 ± 7.3	−1.6 ± 6.7	< 0.001
Final RPE	4.5 ± 2.1	3.5 ± 1.6	< 0.001	8.6 ± 1.7^a^	4.3 ± 2.6	3.3 ± 1.7	< 0.001

Abbreviations: *Δ*, delta; 6MWT, six‐minute walk test; \bpm, beats per minute; CPET, cardiopulmonary exercise test; DBP, diastolic blood pressure; HR, heart rate; MIST, modified incremental step test; mmHg, millimeters of mercury; RPE, rate of perceived exertion; SBP, systolic blood pressure.

^a^ Significant difference compared to submaximal exercise tests.

## 4 Discussion

This was the first study to investigate the measurement properties of the MIST for assessing EC in individuals with T2D. Although the findings do not support the MIST as a substitute for CPET to EC assessment in this population, it may serve as an alternative to the 6MWT in settings with limited space.

The EC estimated from the number of steps climbed during the MIST was not consistent with that measured by peak VO_2_ obtained during CPET. According to the COSMIN classification criteria, the correlation observed between EC assessed by the MIST and CPET (*r* = 0.53) was insufficient to support criterion validity (*r* ≥ 0.70). To date, only one study has investigated the correlation between EC estimated from the MIST and that assessed by CPET, reporting a strong correlation (*r* = 0.85) in individuals with moderate to severe asthma (Barbosa et al. 2022). A possible explanation for the lower correlation observed in our study is that the MIST was performed up to 85% of the age‐predicted maximum heart rate. Although this approach enhances safety, it likely reduces the ability to achieve higher cardiovascular responses and prevents maximal physiological stress, thereby limiting the assessment of maximal exercise performance. In studies involving individuals with respiratory diseases (Camargo et al. 2013; De Andrade et al. 2012; Dal Corso et al. 2013; Vieira et al. 2020; Barbosa et al. 2022; King et al. 2023), the MIST was conducted without heart rate limitation, allowing efforts closer to maximal EC, which may explain the higher correlation coefficient reported by Barbosa et al. (2022).

On the other hand, convergent validity was supported by confirming the hypothesis that the number of steps climbed in the MIST would show a moderate positive correlation with the distance covered in the 6MWT (*r* = 0.65; *p* < 0.001). Previous studies investigating the association between the EC estimated from MIST and the 6MWT have reported strong correlations between the number of steps climbed during MIST and the distance covered in the 6MWT in individuals with bronchiectasis (*r* = 0.80; *p* < 0.001) (Camargo et al. 2013) and COPD (*r* = 0.82; *p* < 0.001) (King et al. 2023). The ICC value observed in our study (ICC: 0.64; 95% CI: 0.44–0.78) is below the COSMIN classification criteria for confirming test‐retest reliability (ICC ≥ 0.70). This result indicates that the MIST did not show consistent EC assessments at different times in individuals with T2D, even though the test and re‐test were performed under the same conditions. Barbosa and colleagues (Barbosa et al. 2022) evaluated individuals with asthma and found an adequate ICC (ICC: 0.88; 95% CI: 0.80–0.93), conducting the test and re‐test on the same visit. Two other studies also reported an adequate ICC in individuals with COPD (ICC: 0.99; 95% CI: 0.97–0.99) (Dal Corso et al. 2013) and bronchiectasis (ICC: 0.97; 95% CI: 0.90–0.99) (Camargo et al. 2013), both conducting the test and re‐test on different visits, which may have contributed to higher ICC values. No floor or ceiling effects were observed. There was also no learning effect, suggesting that a single execution of the MIST may be sufficient for the EC assessment.

As expected, cardiovascular responses and RPE during CPET were significantly higher than those during submaximal tests, since maximal effort imposes greater cardiac and muscular overload. A recent study involving post‐myocardial infarction patients showed lower HR and RPE during submaximal walking tests, such as the incremental shuttle walking test and the 6MWT, compared to CPET (Lim et al. 2022), supporting our findings. In contrast, studies using the MIST as a maximal test for EC assessment of individuals with pulmonary disease, that is, without HR limits, did not observe HR differences compared with CPET (Dal Corso et al. 2013; Vieira et al. 2020). A possible explanation for the differences in cardiovascular responses and RPE during CPET and MIST observed in our study could be the fact that MIST has been limited to 85% of predicted maximum HR.

The findings of HR response and RPE higher during MIST compared to 6MWT were not unexpected, because stepping up and down requires vertical displacement against gravity, resulting in higher body workload (Dal Corso et al. 2013). Furthermore, at least in healthy individuals, greater levels of muscular effort and dyspnea are expected to be elicited in response to exercises with incremental load compared to those with constant load and longer duration (Kearon et al. 1991), which can be comparable to the MIST and the 6MWT, respectively. Considering that MIST elicited higher cardiovascular responses and perceived exertion than the 6MWT, it may be the most suitable option for prescribing aerobic exercise based on achieved HR.

Although an appropriate sample size calculation was performed for the criterion validity investigation, the sample size in our study was below the COSMIN recommendations, representing a key limitation. It is possible that a larger sample would yield a stronger correlation between EC measured by CPET and MIST, potentially supporting criterion validity, as observed in a study including 50 individuals with asthma (Barbosa et al. 2022). The main strength of our study lies in the evidence that the MIST demonstrates adequate convergent validity with the 6MWT for assessing EC in individuals with T2D. Accordingly, its use may be recommended to assess EC in this population in settings with limited space and/or time, given that it is portable and does not require twice executions as the 6MWT. However, further studies are needed to evaluate its criterion validity in a larger sample, to assess its test‐retest reliability, and to determine the clinically meaningful change in the absolute number of steps climbed in response to interventions aimed at improving EC in this population.

## 5 Conclusion

Our study showed that the MIST demonstrated adequate convergent validity and did not present ceiling, floor, or learning effects. Therefore, MIST may be considered as an alternative to assess EC in individuals with T2D in settings where the 6MWT cannot be performed. However, since it was not possible to confirm its criterion validity, MIST should not be considered as an alternative to substitute CPET for EC assessment in this population.

### 5.1 Implications for Physiotherapy Practice

- Individuals with type 2 diabetes often present reduced exercise capacity, highlighting the need for accessible tools to assess physical performance in this population.
- The Modified Incremental Step Test (MIST) is a simple, inexpensive, and portable submaximal exercise test that can be easily applied in clinical and home settings.
- This study provides new evidence that MIST has the potential to be an alternative assessment tool when 6MWT is not available.

## Funding

This study was partly financed by the Coordenação de Aperfeiçoamento de Pessoal de Nível Superior—Brasil (CAPES)—Finance Code 001.

## Ethics Statement

This study was approved by the ethics committees of both research centers (CAAE 64604022.9.2001.5133 and CAAE 64604022.9.1001.5149).

## Consent

All participants provided written informed consent before data collection.

## Conflicts of Interest

The authors declare no conflicts of interest.

## Data Availability

The data associated with this paper are not publicly available but are available from the corresponding author on reasonable request.
